# The Amorphous Solid Dispersion of Chrysin in Plasdone^®^ S630 Demonstrates Improved Oral Bioavailability and Antihyperlipidemic Performance in Rats

**DOI:** 10.3390/pharmaceutics15102378

**Published:** 2023-09-24

**Authors:** Chenhui Wang, Xiaowei Liu, Ruihan Zhao, Meiqing Yang, Wenqian Liu, Qiuyang Dai, Xiaofeng Bao, Yong Chen, Jun Ma

**Affiliations:** 1 School of Pharmacy, Nantong University, 19 Qixiu Road, Nantong 226001, China; 2 School of Pharmacy, China Pharmaceutical University, 639 Longmian Avenue, Nanjing 211198, China; 3 Shenzhen Wanhe Pharmaceutical Company & Guangdong Provincial Key Laboratory of Microecological Preparations, 7 Huitong Road, Shenzhen 518107, China

**Keywords:** chrysin, solid dispersion, XRD, dissolution, stability, in-situ perfusion, oral bioavailability, antihyperlipidemic performance

## Abstract

Chrysin is a flavonoid with various biological activities. However, its low water solubility and strong metabolism render its oral bioavailability rather poor. This study aimed to develop a stable solid dispersion formulation of chrysin to improve the dissolution of chrysin, so as to increase its oral bioavailability and improve its antihyperlipidemic activities. A solid dispersion of chrysin was prepared using a solvent evaporation method, with Plasdone^®^ S630 as the hydrophilic carrier. The formulations were characterized via X-ray diffraction, in vitro dissolution studies, and stability studies. An in-situ perfusion model was used to evaluate the absorption rates. Plasma pharmacokinetics and antihyperlipidemic performance after the oral administration of the chrysin formulations were investigated in rats. It was found that the solid dispersion of chrysin prepared using the drug–polymer mass ratio of 1:6 can form the optimized formulation. X-ray diffraction results showed that the chrysin was in an amorphous state in this optimized formulation. The cumulative release percentage of the optimized solid dispersion of chrysin at pH 1.2 and pH 6.8 was elevated to above 90% within 24 h, indicating that the formulation could enhance the dissolution rates of chrysin. Stability studies showed that the optimized formulation presented acceptable long-term storage stability, but it was susceptible to high temperature and humidity. The solid dispersion of chrysin showed higher absorption rates in the in-situ perfusion model. Pharmacokinetic studies revealed that *C*_max_ and AUC after the intragastric administration of solid dispersion of chrysin were appreciably higher than those resulting from chrysin suspension. The oral bioavailability of the solid dispersion of chrysin was 41 times higher than that of chrysin suspension. Pharmacological studies suggested that the solid dispersion of chrysin was more powerful than chrysin raw material in improving biochemical indicators in the hyperlipidemic model in rats. This study reveals the potential use of a novel oral formulation of chrysin to reduce the currently required high dose.

## 1. Introduction

Hyperlipidemia (HLD) is characterized by high levels of serum total cholesterol (TC), triglyceride (TG), low-density lipoprotein cholesterol (LDL-C), and low levels of high-density lipoprotein cholesterol (HDL-C) in the blood. HLD results from abnormal lipid metabolism, which may lead to fat deposits in artery walls, initiating complications relating to the circulation system, such as atherosclerosis. Moreover, HLD is usually accompanied by fatty liver and obesity, which can further contribute to a series of chronic diseases caused by the dysregulation of sugar/lipid metabolism [1]. Given these risk factors, a reduction in serum lipid levels is a necessary approach that can be used to prevent the formation of these metabolic syndromes.

Phenolic compounds extracted from herbal medicines and natural foods usually present significant activities against lipid metabolic disorders, while little or no adverse side effects were observed in long-term use [2]. As one of the most important classes of phenolic compounds with a wide range of biological activities, flavonoids are recognized as potential therapeutics to effectively ameliorate HLD [3]. The intervention of HLD by flavonoids is principally based on their antioxidative and anti-inflammatory effects in the body [4].

**Figure 1:**
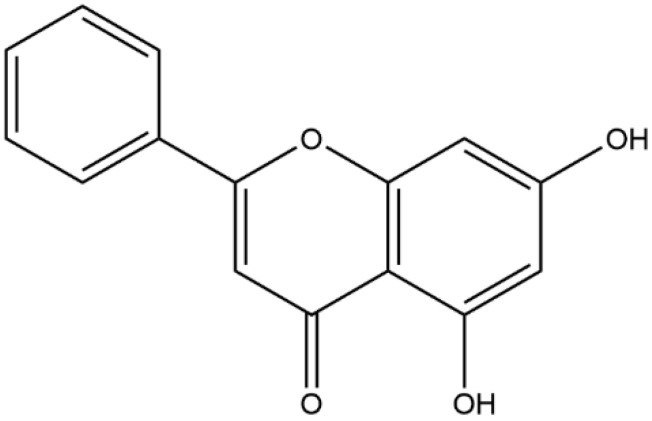
The chemical structure of chrysin (CHR).

Chrysin (CHR, Figure 1), a naturally occurring flavonoid found in diets and functional food (e.g., honey, propolis), has been proven to exhibit a variety of pharmacological functions, such as neuroprotective, hepatoprotective, cardioprotective, antidiabetic, antihyperlipidemic and anticancer effects [5]. Previous studies showed that CHR exhibited hypoglycemic properties in type 2 diabetic rats by insulin sensitization via PPAR-γ activation [6]. CHR also presented an ameliorative effect on HLD complications by modifying fatty acid metabolism due to glycolysis metabolite diminution [7]. Nevertheless, it should be noted that the biological activities of CHR and the pharmacological mechanisms underlying these activities were mainly determined using cell models in-vitro or animal models in-vivo. The results of the current clinical trials have been largely negative. In the human field, CHR has been claimed to be useful in bodybuilding and preventing the conditions such as anxiety, inflammation, gout, etc., but there is no good clinical evidence to support these uses. Currently, dietary supplements containing CHR with the strength of 500 mg, which is quite high, are marketed in the United States.

The aforementioned good in-vitro activities but poor clinical benefits could be attributed partly to the biopharmaceutical properties of CHR. It was suggested that the very poor aqueous solubility [8], extensive pre-systemic metabolism via intestinal and hepatic sulfation and glucuronidation, and efficient excretion through efflux transporters including BCRP and MRP2 [9] are the major reasons resulting in poor systemic bioavailability and hence subtherapeutic systemic exposure for CHR [10,11]. To date, a few of studies have been focused on developing new delivery systems to increase the CHR solubility and improve its therapeutic efficiency. The technologies used included cyclodextrin inclusion [8], mixed micelles [10], liposomes [12], and polymeric nanocapsules [13].

Amorphous solid dispersion (SD) is a solid dispersion in which the drug is evenly dispersed within an excipient matrix in an amorphous form. The amorphous state of the drug in SD is critical for increasing the solubility of poorly water-soluble drugs given the fact that almost no energy is required to break the drug crystal lattice. Moreover, SD is also known to result in higher membrane flux due to a higher supersaturation. Therefore, SD is one of the most attractive approaches to improve the oral bioavailability of drugs classified into BCS II and III [14]. SD technology has been used to improve the solubility and bioavailability of flavonoids. For example, the solubility of luteolin was increased about 250 times after formulating the polyvinylpyrrolidone K40-luteolin SD without changing its physical stability and activity [15]. Naringenin SD had superior water solubility and gastrointestinal absorption relative to native naringenin and showed higher analgesic efficacy in rats than crystalline naringenin [16]. The SD of CHR was previously reported using polyoxyethylene (4) lauryl ether (Brij^®^L4) and aminoclay as the hydrophilic carriers, showing significantly improved dissolution rate and extent of drug release [17]. However, neither of these two excipients has been approved for oral formulation by any regulatory agency.

The purpose of this study was to prepare the SD of CHR (CHR-SD) using an FDA-approved excipient, Plasdone^®^ S630 (PL-S630), and examine its ability to improve oral bioavailability in rats. A solvent evaporation method was used to prepare CHR-SD with different types of carriers, while CHR raw material and physical mixture were used for comparison. The physical state of CHR was characterized using powder X-ray diffraction, and the dissolution profiles of different CHR formulations were assessed. The stability of CHR-SD was monitored in accelerated conditions. The in-situ absorption properties were studied using an intestinal perfusion model, and the pharmacokinetics of CHR after the oral administration of CHR-SD were also investigated in rats. Last but not least, the antihyperlipidemic performance of CHR-SD was examined on an animal model of hyperlipidemia.

## 2. Materials and Methods

### 2.1. Materials

CHR (purity > 99%) was purchased from DASF Biotechnology (Nanjing, China). Plasdone^®^ S630 (PL-S630) was obtained from Ashland (Wilmington, NC, USA). Tween 80 was purchased from Xilong Chemicals (Shantou, China). HPLC-grade methanol and acetonitrile were supplied by Merck KGaA (Darmstadt, Germany). All aqueous solutions were prepared using Milli-Q^®^ water (resistivity > 18 MΩ·cm). All other chemicals used were at least of analytical grade.

### 2.2. Solubility Studies

The solubility of CHR from the CHR raw material was investigated in water, acid (0.1 M HCl), and phosphate buffers (pH 4.5, pH 6.8, and pH 7.4). In order to ensure the sink condition in dissolution studies, the solubility of CHR in water and different phosphate buffers containing 0.5%, 1%, and 2% (*w*/*v*) Tween 80 was also investigated. Briefly, to a flask containing 20 mL of water, excessive amounts of CHR raw material were added and stirred at 400 r/min using a magnetic bar for 24 h at 37 °C, respectively. An aliquot (1 mL) was withdrawn and centrifuged at 10,000 r/min for 10 min. The supernatants were filtered through membrane filters (pore size: 0.22 μm), and the filtrates were subjected to analysis using HPLC-UV. CHR solubility in organic solvents was also investigated as described above. The solubility studies were conducted in triplicates.

The quantification of CHR concentrations in the samples obtained from in vitro studies was carried out on a high-performance liquid chromatography, equipped with an LC-10AT VP pump and an SPD-10A VP ultraviolet–visible detector (HPLC-UV, Shimadzu Corporation, Kyoto, Japan). Isocratic separation was performed using a 150 mm × 4.0 mm Diamonsil^®^ column packed with 5 µm C18 end-capped silica reversed-phase particles. The mobile phase consisted of acetonitrile/0.1% phosphoric acid solution (55/45, *v*/*v*) with a flow rate of 1.0 mL/min. The column temperature was kept at 30 °C, and the injection volume was 20 µL. The detection wavelength was set at 211 nm. Data were collected and processed using the Labsolution workstation. The method for the quantification of CHR was linear over a range of 0.2~100.0 µg/mL, and it was validated with three replicates at 0.2, 0.5, 20, and 75 µg/mL, showing good precision and accuracy for both intra-and interday analyses. The limit of detection (LOD) of CHR, defined as 3 times over the baseline noise, was 50 ng/mL. The limit of quantitation (LOQ) of CHR, defined as 10 times over the baseline noise, was 0.2 µg/mL.

### 2.3. Preparation of CHR-SD

CHR-SD was prepared using the solvent evaporation method. Briefly, 0.5 g of CHR was dissolved in a mixed solvent containing 100 mL of absolute acetone and 25 mL of absolute ethanol via ultrasonication. To the transparent CHR solution, 1.5, 2.0, 2.5, 3.0, or 3.5 g of PL-S630 powders were added and dissolved through magnetic stirring, and the mass ratios between CHR and PL-S630 were 1:3 (CHR 25 wt%), 1:4 (CHR 20 wt%), 1:5 (CHR 16.7 wt%), 1:6 (CHR 14.3 wt%), or 1:7 (CHR 12.5 wt%), respectively. The organic solvent was removed through rotary evaporation. The residual solid was pulverized in a mortar and stored in a desiccator for 48 h before passing through a 100-mesh sieve (pore size, 0.15 mm) to yield the final product. The physical mixture (PM) of CHR and PL-S630 was prepared by mixing accurately weighed CHR powder and PL-S630 (mass ratio, 1:6, CHR 14.3 wt%). The PM was also sieved before further evaluation using the 100-mesh sieve. All products were stored in a desiccator, away from light and humidity, until use.

### 2.4. Characterizations of CHR-SD

#### 2.4.1. X-ray Diffraction (XRD)

Appropriate amounts of CHR raw material, PL-S630, PM (CHR 14.3 wt%), and CHR-SD formulations with different CHR–polymer ratios were subjected to XRD analysis (SmartLab SE, Rigaku, Tokyo, Japan), respectively. The measuring voltage was 40 kV, and the current was 40 mA. The scanning was carried out at a rate of 2°/min within the scanning angle ranging from 3° to 40°.

#### 2.4.2. Dissolution Profiles

In vitro dissolution studies were performed in 900 mL of 0.5% Tween 80 solution (pH 1.2 and pH 6.8) at 37 ± 0.5 °C using the paddle method (100 r/min) in accordance with the USP Apparatus II for dissolution study. First, 10 mg of CHR raw material, PM powder containing 10 mg of CHR, or CHR-SD powder containing 10 mg of CHR was added into a hard gelatin capsule (size 0). The capsule was placed into a basket sinker and then on the bottom of the dissolution vessel. At predetermined time intervals, 2 mL of sample was withdrawn from each dissolution medium. Meanwhile, an equal volume of the isothermal fresh dissolution medium was supplemented. The samples were filtered through membrane filters (pore size, 0.22 µm), and the subsequent filtrates were subjected to analysis using HPLC-UV. The cumulative release of CHR was calculated accordingly. The experiments were performed in sextuplicate.

#### 2.4.3. Stability Studies

Chemical and physical stability studies of CHR-SD (CHR 14.3 wt%) were conducted under three accelerated conditions.

In the first experiment, CHR raw material and CHR-SD were placed in light-tight glass vials at 80 °C for 3 days. The vials were stored in a constant-temperature incubator.

In the second experiment, CHR raw material and CHR-SD were placed in light-tight glass vials and stored in a desiccator that contained a saturated solution of sodium chloride to generate a relative humidity of 75% RH (40 °C) for 1 week. The stored CHR-SD was dried using phosphorus pentoxide for at least 24 h prior to further analysis.

In the third experiment, CHR raw material and CHR-SD were sealed in light-tight glass vials and stored at 25 °C for 3 months.

To examine possible excipient-induced chemical degradation, CHR contents in different CHR samples were determined after the stability studies. Briefly, 35 mg of powder was withdrawn from each sample and dissolved in 10 mL of methanol. The methanolic solution was filtered through a membrane filter (pore size, 0.45 µm), and the subsequent filtrates were subjected to analysis via HPLC-UV. CHR content was calculated by dividing the measured concentration of CHR by the theoretical concentration of CHR.

In the next step, to investigate possible changes in the physical state of CHR molecules in the dispersion, comparisons of the dissolution profiles between the freshly prepared CHR-SD with the samples that had undergone stability studies were conducted using the same dissolution method described in Section 2.4.2.

### 2.5. In Situ Intestinal Perfusion Experiment

Male Sprague Dawley rats (B.W. 220 ± 20 g) were supplied by the Laboratory Animal Center of Nantong University. All the animals were in a 12 h dark–light cycle animal facility with controlled temperature and humidity and had free access to regular chow and water until one day before the animal experiments. The study was approved by the Laboratory Animal Ethics Committee of Nantong University. All animal experimental protocols were performed according to the NIH (National Institutes of Health USA) (2011) Guide for the Care and Use of Laboratory Animals (protocol code S20210317-501).

The rats were fasted for 18 h, anesthetized via the intraperitoneal administration of 1% (*w*/*v*) pentobarbital sodium (5 mL/kg), and placed supine on a surface under a surgical lamp in order to maintain the temperature of the experimental space at about 37 °C. The abdominal cavity was cut with a midline incision, and the intestine was exposed. Two glass tubes (outer diameter 5 mm, inner diameter 3 mm) were inserted through small slits at the duodenum (2 cm from the pyloric sphincter) and ileum ends (immediately proximal to the cecum). Initially, the intestinal segment was rinsed with isotonic saline (37 °C) to clear the gut until the outlet solution was clear; then, PBS solution (pH 7.4) was circulated for approximately 20 min at 5 mL/min using a constant-flow pump (BT100-2J/YZ1515x Peristaltic Pump, Baoding Longer Precision Pump Co., Ltd., Hebei, China). The remaining perfusion was completely expelled by air. The cannula was then connected to two plastic tubes that were connected to the constant-flow pump. PM (CHR 14.3 wt%) and CHR-SD (CHR 14.3 wt%) (equivalent amount to 25 μg/mL of CHR), dissolved in 100 mL of PBS (pH 7.4), were circulated through the gut at 2 mL/min. At 0, 0.5, 1, 2, 3, and 4 h, 1.0 mL of perfusion solution was withdrawn from the reservoir for the quantification of CHR using HPLC-UV, and an equal volume of fresh PBS was supplemented into the circulation in the meantime. The experiment was performed in sextuplicate.

### 2.6. Plasma Pharmacokinetics

Plasma pharmacokinetics after intragastric administration of CHR-SD solution or CHR suspension were investigated in Sprague Dawley rats (B.W. 220 ± 20 g). The rats were supplied and pre-treated as described in Section 2.5. To prepare the CHR-SD solution, an appropriate amount of CHR-SD (CHR 14.3 wt%) was dispersed into water to obtain a solution with the final CHR concentration at 6 mg/mL. To prepare the CHR suspension, an appropriate amount of CHR raw material was dispersed into a CMC-Na solution (0.5%) to obtain a suspension with the final CHR concentration at 6 mg/mL. The two liquid formulations were prepared immediately before the administration.

The rats were fasted overnight (12 h) before the experiments but were allowed free access to water. The two formulations at the dose of 15 mg/kg (calculated for CHR) were administered intragastrically into randomly grouped rats (*n* = 6), respectively. Blood samples withdrawn from the orbital venous plexus were collected into heparinized tubes at 0, 0.25, 0.5, 0.75, 1, 2, 3, and 6 h after administration. Plasma was immediately separated via centrifugation at 4000 r/min for 10 min and then stored at −20 °C.

The quantification of CHR plasma concentrations was performed on an Acquity^®^ ultraperformance liquid chromatography system coupled with a Quattro Premier XE tandem mass spectrometer (UPLC-MS/MS, Waters Corporation, Milford, CT, USA). The chromatographic separation was achieved using an Acquity^®^ UPLC BEH C18 column (50 mm × 2.1 mm, 1.7 µm). The mobile phase consisted of 0.1% formic acid in methanol/0.1% formic acid in water (60/40, *v*/*v*) with a flow rate of 0.2 mL/min. The column temperature was kept at 40 °C, and the injection volume was 5 µL. For detection using mass spectrometry, electrospray ionization was set at a negative-ion mode (ESI-), and multiple reaction monitoring modes (MRMs) were employed for quantitative analysis with the parent–daughter ion transitions of *m*/*z* 252.90 → 143.10 for CHR and *m*/*z* 270.90 → 150.90 for internal standard (naringenin, IS). The capillary voltage was 3.5 kV, and the ion source temperature was 210 °C. Nitrogen was used as the nebulizer gas and auxiliary gas, and the flow rates were 400 L/h and 45 L/h, respectively. Argon was used as collision gas. Data processing was performed using Waters MassLynx 4.1 software. A stock solution of CHR (0.1 mg/mL) was prepared by dissolving an appropriate amount of CHR into methanol. The stock solution was further diluted with methanol to obtain a series of working solutions with CHR concentrations of 400, 200, 100, 40, 20, 10, 4, and 2 ng/mL. The solution of IS (100 ng/mL) was prepared in methanol as well. First, 100 µL of each CHR working solution and 50 µL of IS solution were mixed with 50 µL of blank rat plasma to obtain a series of standard solutions with the final CHR concentrations of 200, 100, 50, 20, 10, 5, 2, and 1 ng/mL, respectively. The standard solutions were mixed using a vortex and then centrifuged at 10,000 r/min for 10 min. The separated supernatants were subjected to UPLC-MS/MS analysis. The method for the quantification of CHR was linear over a range of 1 ~ 200 ng/mL and was validated with three replicates at 1, 5, 50, and 200 ng/mL, showing good precision and accuracy for both intra- and interday analyses. The LOQ and LOD were 1 and 0.2 ng/mL, respectively.

Prior to the formal analysis of the plasma samples, 50 μL of the freshly thawed plasma sample was mixed with 100 μL of methanol and 50 μL of IS solution, mixed on a vortexer, and centrifuged at 10,000 r/min for 10 min. The supernatants were subjected to UPLC-MS/MS analysis as described above.

All pharmacokinetic parameters, including maximum plasma concentration (*C*_max_), time to reach the maximum plasma concentration (*T*_max_), and area under the blood concentration–time curve (AUC), were calculated using the DAS 3.0 software (BioGuider Medicinal Technology, Shanghai, China) using the non-compartmental model.

### 2.7. Antihyperlipidemic Performance

Experiments were performed on four groups of Sprague Dawley rats (220 ± 10 g, *n* = 6), which were supplied and pre-treated as described in Section 2.5. The control group was fed a normal control diet with distilled water during the whole period of the experiments. The HFD group (model group), CHR suspension group, and CHR-SD group were fed a high-fat diet (HFD), which was prepared by supplementing a normal control diet with 20.0% sucrose, 15% lard oil, 1.2% cholesterol, 0.2% sodium cholate, 1.2% calcium hydrogen phosphate, 0.8% stone powder, and 5.0% casein. At the end of the eight weeks of feeding with the HFD, the serum lipid profile was measured to ascertain the induction of hyperlipidemia. The hyperlipidemic rats were then fed HFD for another 1 month but were treated with different formulas daily during this period: the HFD group was given distilled water daily by oral gavage (10 mL/kg), whereas the CHR suspension and CHR-SD groups were given CHR suspension and CHR-SD solution (prepared as described in Section 2.6), respectively, via intragastric administration, carried out daily at a dose of 15 mg/kg (calculated for CHR) for both. After 1 month of treatment, the animals were fasted for 12 h, and then blood samples were withdrawn from the orbital venous plexus. Plasma was immediately separated via centrifugation at 4000 r/min for 10 min and then stored at −20 °C. The rats were then anesthetized and sacrificed using cardiac puncture. Their livers and white adipose tissues (WAT, from inguinal and epididymal) were excised, rinsed in normal saline, and weighed, and their indexes were calculated: liver or WAT index = weight of wet liver or WAT/body weight × 100%. The liver samples were then immediately frozen in liquid nitrogen and stored at −80 °C for further investigations into the related biochemical markers.

Serum concentrations of total cholesterol (TC), triglyceride (TG), low-density lipoprotein cholesterol (LDL-C), and high-density lipoprotein cholesterol (HDL-C) were determined from plasma samples using commercial assay kits according to the instructions provided by the manufacturer (Nanjing Jiancheng Bioengineering Institute, Nanjing, China). The activities of hepatic superoxide dismutase (SOD) and catalase (CAT), as well as the levels of hepatic malondialdehyde (MDA), were determined from liver samples using commercial assay kits according to the instructions provided by the same manufacturer.

### 2.8. Statistical Analysis

Data are expressed as the mean ± SD. Outliers determined using the Dixon test were discarded. The results were evaluated statistically using either Student’s *t*-test for analysis containing two groups or ordinary one-way analysis of variance (ANOVA) with Tukey’s multiple-comparison test (GraphPad Prism, Version 8.0.2) for analysis containing three or more groups. The level of significance was determined with *p* values as follows: *p* > 5 × 10^−2^ was considered to indicate a non-significant difference; 1 × 10^−2^ < *p* < 5 × 10^−2^ was considered to indicate a significant difference; and *p* < 1 × 10^−2^ was considered to indicate a very significant difference.

## 3. Results and Discussion

### 3.1. Solubility Studies

The high crystallinity renders the water solubility of CHR extremely poor, under the low level of quantification, using the HPLC-UV method (<0.2 µg/mL). CHR presents two phenol hydroxyl groups in its structure (Figure 1), with a calculated first ionization constant (p*K*_a1_) value of 6.65 [18], showing that CHR is a very weak acid with a pH-dependent solubility, theoretically. However, it was still not able to obtain the saturated solubility of CHR in acid (0.1 M HCl) or phosphate buffers (pH 4.5, pH 6.8, and pH 7.4) using the HPLC-UV method. Therefore, more efforts should be made to find an appropriate solvent system to meet the sink condition prior to the dissolution studies. Given the extremely poor intrinsic aqueous solubility, the addition of surfactant to increase the CHR solubility was tested. It was found that the addition of 0.5%, 1%, or 2% Tween 80 in water increased the CHR solubility to detectable levels (16.3, 30.9, or 70.3 μg/mL, respectively). In the presence of the surfactant, the change in the pH value still did not significantly affect the solubility of CHR: the solubility of CHR in hydrochloride solution (pH 1.2) and phosphate-buffered solution (pH 6.8) containing 0.5% Tween 80 was 18.89 ± 0.17 and 16.90 ± 0.26 μg/mL, respectively. Therefore, the 0.5% Tween 80 aqueous solution with pH 1.2 and pH 6.8 was selected as the release medium in subsequent in-vitro dissolution studies.

### 3.2. Preparation of CHR-SD

In the preliminary studies, none of the attempts to prepare CHR-SD by dissolving CHR into various solvents without the addition of the excipient and then performing rotary evaporation were successful, indicating that CHR is a rapid crystallizer [19]. Therefore, it is essential to find a proper crystallization inhibitor in order to ensure the stability of the SD in terms of crystallization in the solid state, during preparation and storage, and to maintain a supersaturated state when the drug releases from its SD formulation into the aqueous media of gastrointestinal tract.

Various polymers have been proven effective in improving the physical stability of amorphous solids by inhibiting the crystallization of the amorphous form [20]. Copovidone is a synthetic random copolymer consisting of *N*-vinyl-2-pyrrolidone and vinyl acetate in 60:40 ratios. The pyrrolidone ring is responsible for water solubility and solubilization properties, while the vinyl acetate monomer reduces glass transition temperature and hygroscopicity, compared with homopolymers of pyrrolidone. The unique physicochemical properties of Plasdone-grade copovidone make it attractive in the use of matrix polymer for SD formulation to enhance the solubility and bioavailability of poorly soluble drugs [21]. For example, PL-S630-based tadalafil solid dispersion was designed to enhance the dissolution rate [22]. PL-S630-based indomethacin solid dispersion showed a significant ability to enhance solubility, prevent recrystallization during storage, and facilitate the dissolution of the poorly water-soluble indomethacin [23]. The mechanism underlying the solubility and stability enhancement is attributed to specific interactions (e.g., hydrogen bonding) between the polymer and the drug [24,25]. Therefore, PL-S630 was used as the hydrophilic carrier for producing CHR-SD. In order to investigate the effect of drug loading on the physical state of the drug in the SD, five CHR-SD formulations were prepared with the weight percentages of CHR spanned from 12.5% to 25%, using the solvent evaporation method, which is suited for the lab-scale production of SD.

The solvent for the preparation of CHR-SD was selected by investigating the saturated solubility of CHR raw material within a series of organic solvents, among which acetone showed the highest solubility to CHR (5 603.4 ± 476.1 μg/mL); therefore, acetone was selected as the principal solvent for CHR. Given the low boiling point of acetone (56 °C), which might lead to phase separation during SD preparation and hence fail to obtain a homogeneous and stable amorphous SD due to the fast evaporation of acetone under vacuum, another more polar solvent, ethanol, with a higher boiling point (78 °C) and a moderate solubility to CHR (3 305.1 ± 46.9 μg/mL), was chosen as the co-solvent with acetone (*V*_acetone_:*V*_ethanol_ = 1:1). This mixed solvent system was previously used in preparing the SD of another polyphenol active compound, resveratrol [26].

### 3.3. Characterizations of CHR-SD

#### 3.3.1. XRD

**Figure 2:**
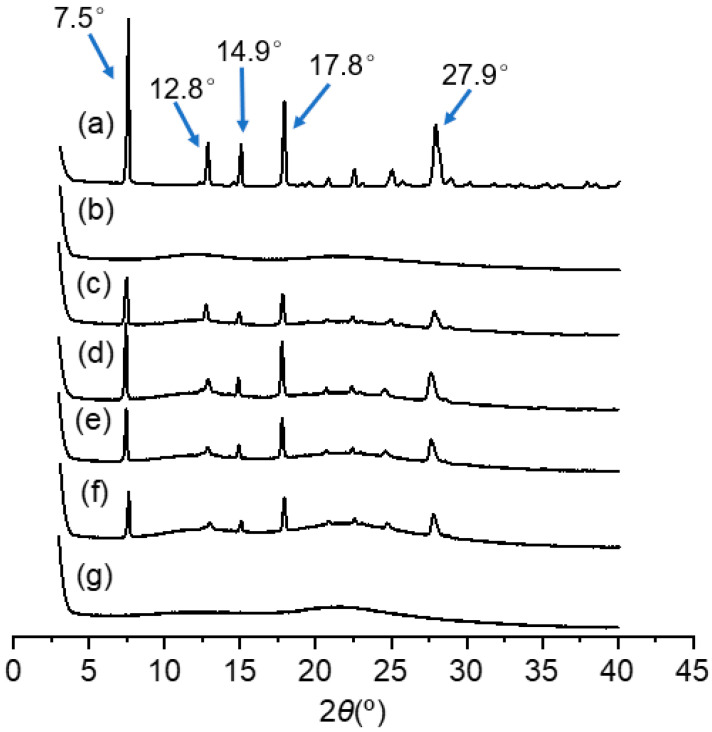
X-ray diffraction patterns: (**a**) CHR raw material; (**b**) PL-S630; (**c**) PM; (**d**) CHR-SD (CHR 25 wt%); (**e**) CHR-SD (CHR 20 wt%); (**f**) CHR-SD (CHR 16.7 wt%); (**g**) CHR-SD (CHR 14.3 wt%).

The characterizations of CHR-SD formulations were first carried out by analyzing XRD patterns. As shown in Figure 2, the representative diffraction peaks of crystalline CHR appeared at 7.5°, 12.8°, 14.9°, 17.8°, and 27.9° in the CHR raw material, demonstrating its high crystallinity, which is consistent with a previous study [27]. In comparison, the patterns of the carrier showed no peaks at all, indicating its amorphous nature. Comparing the height of the peaks in the PM with those in the CHR raw material, the reduced magnitude of peaks was observed in the former, which should be attributed to the dilution effect of the carrier. Similarly, a reduction was observed in the height of the peaks in the XRD patterns of the CHR-SD formulations (CHR 16.7~25 wt%), pointing to a decrease in CHR crystallinity in these formulations. The absence of major peaks in XRD patterns was detected when the percentage of CHR in CHR-SD was reduced to 14.3 wt%, confirming the transformation of the crystalline polymorph of CHR into its amorphous polymorph in the form of SD. These conclusions from XRD analysis were further explored with dissolution studies.

#### 3.3.2. Dissolution

**Figure 3:**
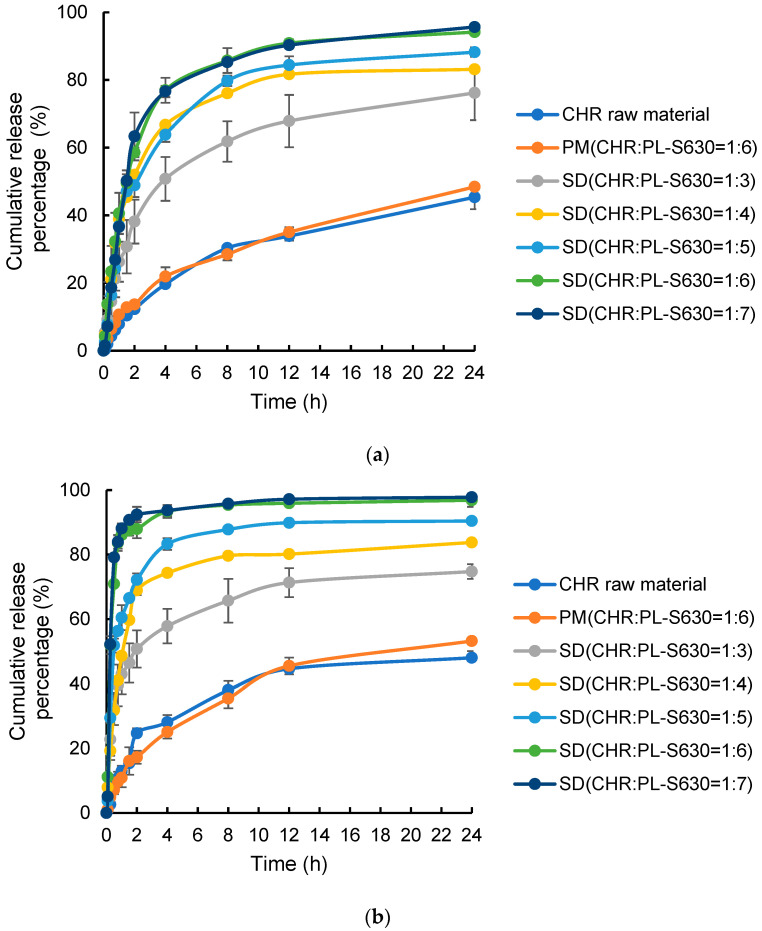
Dissolution profiles of different CHR samples in (**a**) 0.5% Tween 80 aqueous solution at pH 1.2 and (**b**) 0.5% Tween 80 aqueous solution at pH 6.8 (*n* = 6).

It is worth noting that the amount of CHR (raw material, PM, or CHR-SD) added to the dissolution vessel (900 mL) was 10 mg, which corresponded to a maximum CHR concentration of 11.1 μg/mL in the dissolution solution. This concentration was much lower than the saturated solubility of CHR when 0.5% Tween 80 was used (see Section 3.1). As shown in Figure 3, the dissolution profiles between the CHR raw material and PM almost overlap at 24 h in both dissolution media, suggesting that PL-S630 did not enhance the dissolution of crystalline CHR raw material. In contrast, formulating CHR into solid dispersion using PL-S630 as the hydrophilic carrier significantly enhanced the dissolution of CHR. For example, when the ratio of CHR to PL-S630 changed from 1:3 to 1:5, the cumulative dissolution percentages for 4 h were about 50~65% at pH 1.2 and about 60~80% at pH 6.8, which were significantly higher than those from the CHR raw material and PM within the same time period (about 20~25%). Moreover, the increased ratio of PL-S630 for preparing CHR-SD led to faster release rates in the early stages and higher cumulative release percentages in the final time point in both dissolution media. For example, when the ratio of CHR to PL-S630 was 1:6, the cumulative release percentages within 4 h were ~76% and ~93% in pH 1.2 and 6.8 environments, respectively, and approximately 95% in both environments within 24 h, which were significantly higher than the release percentages within the corresponding time points when using any CHR-SD with lower PL-S630 contents. The cumulative release from CHR-SD (CHR:PL-S630 = 1:7) was very close to the ratio at 1:6 without significant differences at almost all time points, suggesting that a further increase in the proportion of PL-S630 was not helpful in dissolution enhancement. The variations observed in cumulative release percentages in different curves may suggest that the capacity of PL-S630 to stabilize the amorphous state of CHR was sufficient only if the mass ratio of CHR to PL-S630 reached 1:6 (CHR 14.3 wt%); lower percentages of PL-S630 would probably result in microcrystalline particles in the dissolution media and render the dissolution behaviors more complicated.

#### 3.3.3. Stability

The chemical stability of CHR was first examined. In the first stability study, where the samples underwent high temperature for 3 days, about 26~32% loss of CHR content was recorded in the CHR raw material and CHR-SD, and no statistical significance was observed between them. This result suggests that CHR, as a flavonoid compound, is susceptible to oxidative degradation at high temperatures given its phenolic groups. Compared with the CHR raw material, the formulation of CHR-SD did not further deteriorate the stability of CHR, although the use of copovidone in SD is associated with stability concerns due to the presence of trace amounts of peroxides in copovidone [28]. To improve stability under high-temperature conditions, an antioxidant or peroxide scavenger, such as BHT, might be considered for addition to CHR-SD. In the second stability study, the loss of CHR content was about 7.2% for the CHR raw material and 3.5% for CHR-SD after the study, showing that, under high humidity, CHR was not as sensitive as CHR in high temperatures. In the third study, no significant loss of CHR content was found in the CHR raw material or CHR-SD after 3 months of storage at room temperature without light.

**Figure 4:**
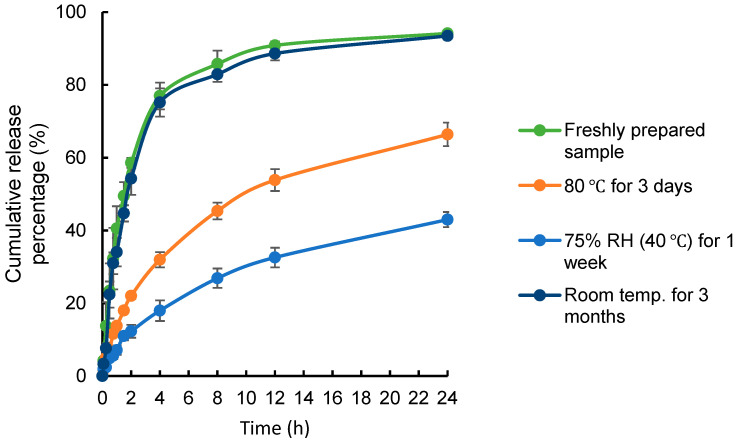
Dissolution profiles of CHR-SD samples after the stability studies, compared with freshly prepared CHR-SD sample, in a 0.5% Tween 80 aqueous solution at pH 1.2 (*n* = 6).

Then, the physical stability of CHR was examined by performing dissolution studies in 0.5% Tween 80 aqueous solution at pH 1.2 and pH 6.8. The corresponding dissolution behaviors for each sample under these two pH values were comparable. The dissolution profiles of CHR-SD subjected to stability studies in 0.5% Tween 80 aqueous solution at pH 1.2 are shown in Figure 4. It was found that the in-vitro dissolution profiles of CHR-SD that had been subjected to the third stability study (room temperature for 3 months) did not significantly change, compared with that from the freshly prepared CHR-SD, suggesting good long-term storage stability. However, the cumulative releases of CHR from the samples obtained from the first (80 °C for 3 days) and second (75% RH (40 °C) for 1 week) stability studies were much lower. It is reasonable to infer that the significant loss of CHR content at high temperatures and the physical transition of CHR from an amorphous state to a crystalline state under high humidity contributed to the reduced cumulative releases obtained from the CHR-SD samples in the first and second stability studies. Therefore, in the storage of CHR-SD, high temperature and humidity should be avoided, as the former principally deteriorates the chemical stability of CHR-SD, while the latter damages its physical stability.

### 3.4. In-Situ Intestinal Perfusion Experiment

**Figure 5:**
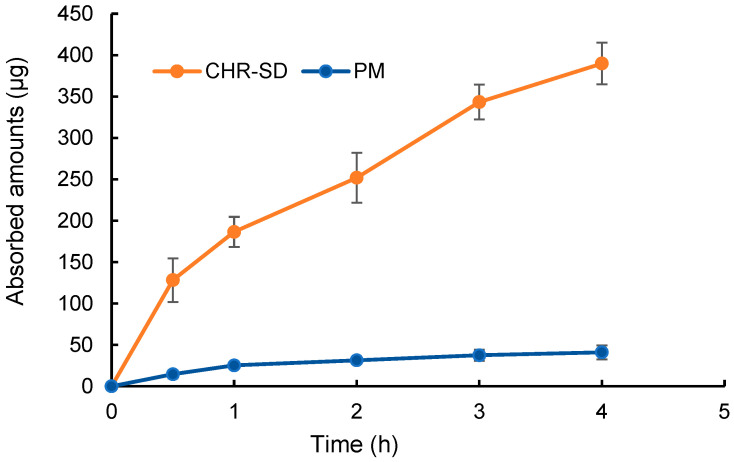
The absorption amount of CHR in the in-situ experiment using the intestinal perfusion of CHR-SD and CHR suspension with the equivalence of 25 μg/mL of CHR (*n* = 6).

The absorption rates of CHR from CHR-SD and PM were examined in-situ using the intestinal perfusion model. As shown in Figure 5, the absorption rates of CHR from CHR-SD and PM were 15.6% and 1.6%, respectively, indicating that the SD could significantly enhance the absorption rate of CHR. The higher absorption rate of CHR-SD can be attributed to the higher concentration of solubilized CHR present in the perfusion fluid. More importantly, as the concentration of CHR in the perfusion fluid of CHR-SD was higher than its saturated solubility, a supersaturated system was established, which could result in higher thermodynamic activities of the molecules. The elevated thermodynamic activity of CHR molecules, which may last for several hours in the gastrointestinal tract, can facilitate CHR to cross the epithelium layer of the intestinal wall, given the driving force to transform an unstable system with higher thermodynamic activity into a stable system with lower thermodynamic activity [29].

### 3.5. Bioavailability Study

**Figure 6:**
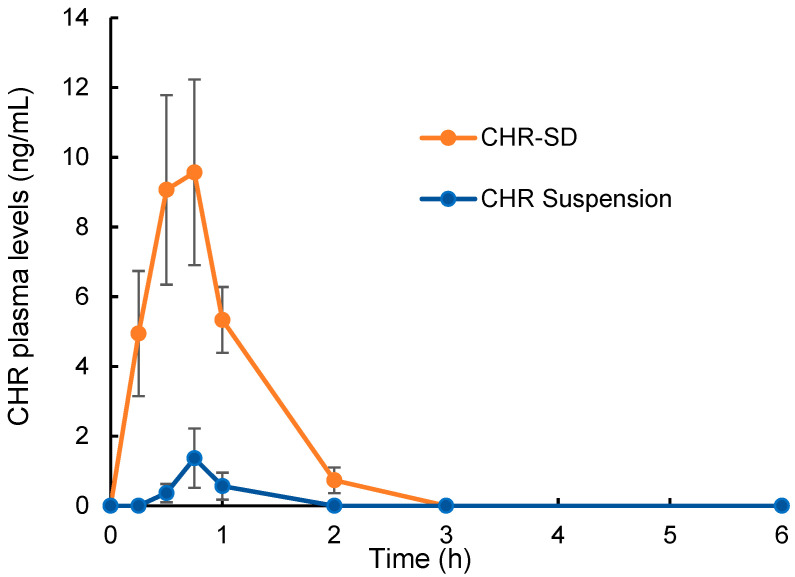
Plasma levels of CHR after intragastric administration of CHR-SD and CHR suspension with the equivalent dose of 15 mg/kg CHR (*n* = 6).

The plasma levels of CHR after the intragastric administration of CHR-SD and CHR suspension are shown in Figure 6, and the pharmacokinetic parameters, namely the maximum plasma concentration (*C*_max_), time to reach the maximum plasma concentration (*T*_max_), and the area under the blood concentration–time curve (AUC_0–6 h_), were calculated with the DAS software (Table 1) using a non-compartmental model. The results show that the plasma concentration of CHR increased rapidly after the oral administration of CHR-SD. In comparison, the oral administration of CHR suspension resulted in a much lower level of *C*_max_. The AUC_0–6 h_ value for CHR-SD was approximately 41 times higher than that of the CHR suspension. These results suggest that CHR-SD enables higher oral bioavailability of CHR, which can reasonably reduce the demanded dose. It should be also noted that, although the dose administered was not too low (15 mg/kg), the plasma levels of CHR after the administration of both formulations were relatively low—less than 10 ng/mL. The low systemic exposure of CHR is not only attributed to the comparatively low absorption rates but also results from the extensive first-pass metabolism. It was reported that CHR can be rapidly metabolized by UGTs to produce chrysin-7-O-glcuruonide as one of the major metabolites [5]. It was also reported that the plasma chrysin sulfate concentrations were 30-fold higher than the parent drug in the human body [11]. Multiple factors limited the oral bioavailability of CHR raw material, including low aqueous solubility, low-to-moderate permeability, and rapid metabolism. However, the formulation used in this study could only increase the aqueous solubility by transforming the crystalline state of CHR into an amorphous state and thus enhance the permeability by establishing a supersaturated system in the gastrointestinal tract. This rapid metabolism may largely be inhibited through the coadministration of UGT inhibitors, such as sodium oleate [30]. This possibility will be investigated in future studies.

### 3.6. Antihyperlipidemic Performance

At the end of the experiment, the hepatic index of the rats in the HFD group (4.23 ± 0.38%) presented a significant increase after the HFD treatment when compared with that in the control group (2.86 ± 0.29%, *p* < 0.01), indicating the occurrence of liver disease in the rats (Table 2). In comparison, the hepatic index of rats in CHR-treated groups markedly decreased, which was significantly lower than that in the HFD group (*p* < 0.05 for both the CHR suspension and CHR-SD groups). The WAT index (Table 2) of the rats in the HFD group (8.46 ± 1.58%) was also significantly higher than that in the control group (3.89 ± 0.51%, *p* < 0.01), whereas significantly lower WAT indices were found in the CHR suspension group (*p* < 0.05) and CHR-SD group (*p* < 0.01), compared with that in the HFD group.

**Figure 7:**
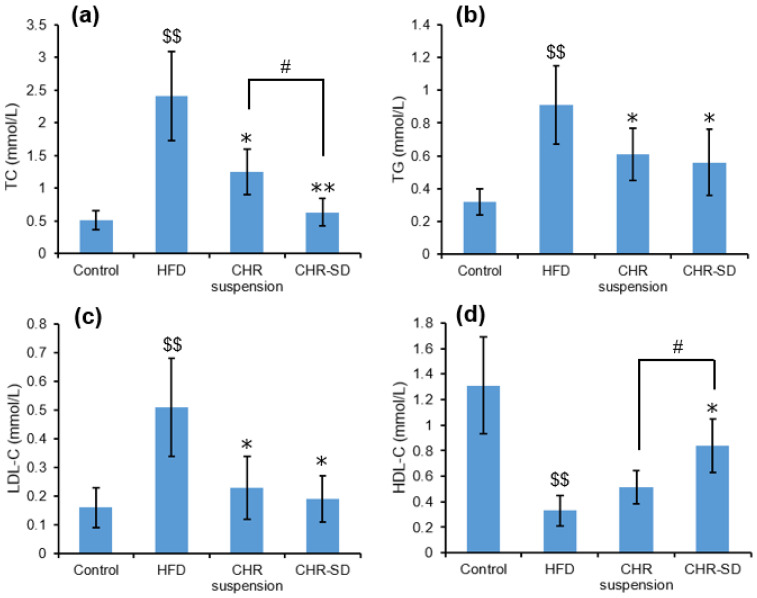
Serum lipid: (**a**) TC (mmol/L); (**b**) TG (mmol/L); (**c**) LDL-C (mmol/L); (**d**) HDL-C (mmol/L). The results are expressed as mean ± standard deviation (*n* = 6). Statistical analysis was performed using ANOVA. The means with different superscripts are considered significantly different. ^$$^ *p* < 0.01, HFD vs. control; * *p* < 0.05, ** *p* < 0.01, HFD vs. other groups; ^#^ *p* < 0.05, CHR-SD group vs. CHR suspension group.

After providing HFD for 8 weeks, TC, TG and LDL-C levels in serum were significantly higher than those in the control group (*p* < 0.05), and HDL-C level was significantly lower (*p* < 0.05), suggesting that the dyslipidemia model was successfully established in rats. Upon the completion of the experiments, these significant differences became larger between these two groups (*p* < 0.01). After administrating CHR suspension or CHR-SD for 1 month, significant decreases in the serum TC, TG, and LDL-C levels were observed in both treated groups, compared with those in the HFD group, which are shown in Figure 7a–c. In the meantime, a significant increase in the serum HDL-C level was observed in the CHR-SD group compared with that in the HFD group (Figure 7d). No statistically significant differences were found between the CHR suspension and CHR-SD groups regarding TG and LDL-C. It was observed that the treatment using CHR-SD was more effective than the treatment using CHR suspension in reducing the level of TC and increasing the level of HDL-C.

**Figure 8:**
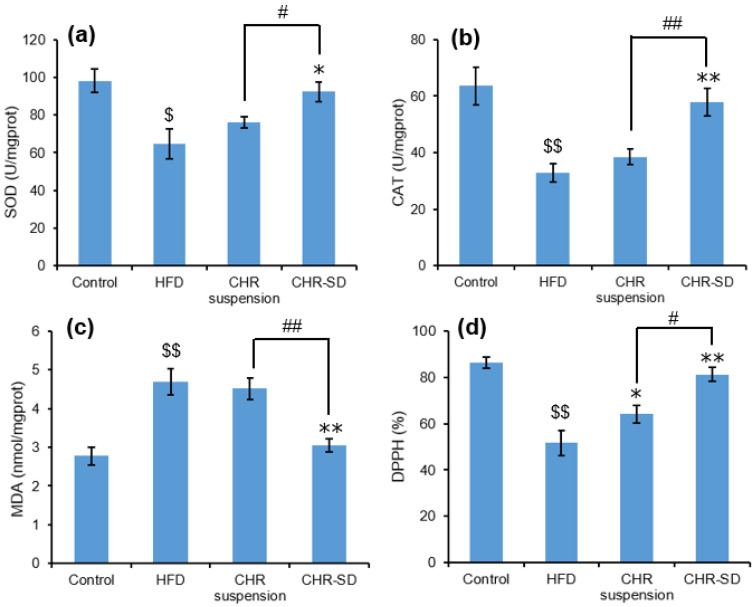
Antioxidative indicators in the liver: (**a**) SOD (U/mgprot); (**b**) CAT (U/mgprot); (**c**) MDA (nmol/mgprot); (**d**) DPPH (%). The results are expressed as mean ± standard deviation (*n* = 6). Statistical analysis was performed using ANOVA. The means with different superscripts are considered significantly different. ^$^ *p* < 0.05, ^$$^ *p* < 0.01, HFD vs. control; * *p* < 0.05, ** *p* < 0.01, HFD vs. other groups; ^#^ *p* < 0.05, ^##^ *p* < 0.01, CHR-SD group vs. CHR suspension group.

Compared with the control group, the HFD group presented significantly lower levels of SOD, CAT, and DPPH antioxidant capacity, in addition to a significantly higher level (*p* < 0.01) of MDA, indicating that serious oxidative damage had occurred in the liver. Compared with the HFD group, the treatment using CHR-SD presented significantly higher levels of SOD, CAT, and DPPH antioxidant capacity, in addition to a significantly lower level of MDA. In comparison, the treatment using CHR suspension was only effective in increasing the level of DPPH antioxidant capacity, suggesting that CHR-SD was more powerful in improving the liver antioxidant capacity of dyslipidemia rats than CHR suspension (Figure 8).

The better antihyperlipidemic performance of CHR-SD in rats should be attributed to the higher absorption rate and improved oral bioavailability of CHR, which again proved that enhancement in the oral bioavailability of poorly water-soluble lipid-lowering drug could effectively improve its pharmacodynamic activity [31]. This could be observed with the CHR dose used, 15 mg/kg, which was used as a “low dose” in previous reports [32,33]. In this paper, we clearly demonstrated the “low dose” of CHR using its solid dispersion formulation was effective. It has also been reported that the polymeric encapsulation of CHR improves the oral bioavailability of the drug and increases its absorption, thus enhancing its antihyperlipidemic properties [13]. Given these results, it can be concluded that the improvement observed in the pharmacokinetic and pharmacodynamic behaviors of CHR after its formulation into solid dispersion using PL-S630 as a hydrophilic carrier makes it a promising and beneficial strategy for lipid-lowering treatment.

HFD-induced hyperlipidemic animal models have been widely used for the evaluation of the lipid-lowering efficacies of drugs and their formulations. It should also be noted that the use of genetically hyperlipidemic animal models can more precisely reflect the essential pathological changes in the patients. Therefore, the developed formulations will be tested using genetically hyperlipidemic animal models in future studies.

## 4. Conclusions

In this study, CHR-SD was successfully developed using a pharmaceutical excipient PL-S630 with the solvent evaporation method. The optimized ratio of CHR to PL-S630 was 1:6, where the CHR molecules were amorphously dispersed in the polymer matrix, determined by the XRD pattern. The optimized formulation presented faster and more complete release in the dissolution media in vitro than the CHR raw material. The optimized CHR-SD presented good long-term storage stability. With higher rates of absorption, CHR-SD successfully resulted in notably higher *C*_max_ and AUC than CHR suspension. CHR-SD also exhibited a more powerful activity to intervene in hyperlipidemia in the rat model in this study. In conclusion, the use of the PL-S630 to formulate CHR-SD leads to an increase in the oral bioavailability of CHR, suggesting the potential use of this novel CHR oral formulation to reduce the currently required high dose.

## Figures and Tables

**Table 1:** Pharmacokinetic parameters of CHR after intragastric administration of CHR-SD and CHR suspension with the equivalent dose of 15 mg/kg CHR (*n* = 6).

Parameters	CHR-SD	CHR Suspension
*T*_max_ (h)	0.62 ± 0.12	0.71 ± 0.09
*C*_max_ (ng/mL)	9.57 ± 2.66 **	1.37 ± 0.85
AUC_0–6 h_ (h·ng/mL)	13.81 ± 2.70 **	0.33 ± 0.18

** *p* < 0.01, compared with CHR suspension.

**Table 2:** Effects of CHR treatment on hepatic indexes (%) and WAT indexes (%) (*n* = 6).

Groups	Hepatic Indexes (%)	WAT Indexes (%)
Control	2.86 ± 0.29	3.89 ± 0.51
HFD	4.23 ± 0.38	8.46 ± 1.58
CHR suspension	3.57 ± 0.34 *	6.22 ± 1.21 *
CHR-SD	3.11 ± 0.21 *	4.47 ± 0.79 **

* *p* < 0.05, ** *p* < 0.01, compared with HFD group.

## Data Availability

All data available are reported in the article.
